# Analysis of the Contribution of Home Gardens to Household Food Security in Limpopo Province, South Africa

**DOI:** 10.3390/su16062525

**Published:** 2024-03-19

**Authors:** Mbalenhle Gwacela, Mjabuliseni Simon Cleopas Ngidi, Simphiwe Innocentia Hlatshwayo, Temitope Oluwaseun Ojo

**Affiliations:** 1 Department of Agricultural Extension and Rural Resource Management, School of Agricultural, Earth and Environmental Sciences, College of Agriculture, Engineering and Science, University of KwaZulu-Natal, Private Bag X01, Scottsville, Pietermaritzburg 3201, South Africa; 2 Centre for Transformative Agricultural and Food Systems, School of Agricultural, Earth and Environmental Sciences, College of Agriculture, Engineering and Science, University of KwaZulu-Natal, Private Bag X01, Scottsville, Pietermaritzburg 3201, South Africa; 3 Department of Agricultural Economics, Obafemi Awolowo University, Ile-Ife 220103, Nigeria; 4 Disaster Management Training and Education Centre for Africa, University of the Free State, Bloemfontein 9301, South Africa

**Keywords:** household food security, home gardening, rural communities, agricultural education

## Abstract

Addressing food security is one of the national priorities in South Africa, enshrined under the country’s constitution, yet there is a growing percentage of households struggling to meet their food requirements. Food insecurity and malnutrition remain severe problems in rural communities and can be addressed through home gardening. This study aimed to assess the contributions of home gardens to food security in Limpopo Province. This study employed a quantitative research methodology. A total of 2043 rural households were selected using multistage stratified random sampling. The Household Food Insecurity Access Scale (HFIAS) was used to measure household food insecurity levels of home garden participants. Results showed that 46% of participants were food secure, 24% were severely food insecure, 17% were moderately food insecure and 13% were mildly food insecure. The results from the endogenous switching Poisson regression model showed that gender, household size, wage/salary, access to land, agriculture-related assistance and market distance had a positive influence on household food security of home garden participants. On the other hand, employment status and receiving any social relief had a negative association with household food security of home garden participants. The results also showed that employment status had a positive influence on the food security of home garden non-participants, while education, access to land, wage/salary and age had a negative influence. The results from average treatment effects (ATEs) showed that households that participated in home garden production had a negative and significant (*p*-value < 0.05) impact on household food insecurity. This study concludes that involvement in home gardening improves food security. Household food security can be enhanced through agricultural training and skills enhancement directed at increasing participation in home gardening in rural areas, thus addressing income and food security challenges. Agricultural education needs to be introduced and facilitated at school levels so that an understanding of food systems, nutrition and food security can be attained from younger age groups.

## 1 Introduction

Globally, there are ongoing considerations for better and more sustainable ways to feed the growing population. By the year 2050, the global population is predicted to reach over 9 billion [1,2]. Some countries will have difficulty meeting the growing demand which could lead to hunger and food insecurity for many households struggling to cope with food price volatilities and local food production limitations. This is the reality for many households within the lower income range that cannot be guaranteed access to sufficient food [3,4]. In 2020, Statistics South Africa recorded that 23.6% of South Africans ranged from moderately to severely food insecure, while 14.9% experienced severe food insecurity [5]. Out of 113 countries, South Africa ranked as the most food secure in the African continent [6]. South Africa is considered as food secure, technically, as it produces enough staple food to feed its population. However, rural households continue to encounter food security difficulties.

Multiple strategies are required to address the growing food production and food insecurity challenge. The success of these strategies relies primarily on existing socio-political conditions, the existing economic climate and available resources [7]. Home food gardening is perceived as a successful strategy that can contribute towards increasing food access. Although home food gardens have evolved over the years as societies go through urbanization, they have been an essential component for families providing their own food all over the world [1,8]. Home food gardens are described as a family-managed microenvironment within a larger farming system that frequently exhibits high levels of species diversity [9–11].

Home food gardens are among the most important sources of food in developing countries, as they significantly contribute to households’ food consumption needs for improved health and nutrition [12,13]. Home food gardening is practiced in most rural areas in South Africa as a source of household income and food consumption [11]. Home garden programs contributed towards a reduction in food insecurity [11]. In addition, home gardens are considered as a community’s most adaptable and accessible land resource that plays a significant role in reducing household vulnerability while ensuring food security [14,15]. They ensure quick access to food that can be harvested, prepared and eaten by family members regularly and with ease. To some extent, they are a production system that can easily be accessed by under-resourced households. Household farming has the potential to provide a variety of foods that can increase the quantity and improve the quality of nutrients for household diets [4,12].

Despite the potential benefits and opportunities that home gardening provides to rural households, there are various factors that can hinder production. These factors include lack of farming skills, availability of space and water, security of tenure, availability of time, and pest and disease occurrence. In addition, there is a lack of planting materials, an inadequate supply of quality seeds [16] and a lack of finance. Other constraints include a lack of access to water, poor soil fertility, limited access to agricultural inputs, limited marketing opportunities, and a lack of information and advisory services [7,14]. These challenges accelerate food insecurity among low-income households as they affect the production of diversified foods.

South Africa established a food security policy enshrined in the Bill of Rights, Section 27, 1 (b), which highlights that every citizen has a right to access to sufficient food and water and that the state must take reasonable legislative and other measures to achieve this [15]. The Food and Nutrition Security Implementation Plan was formulated to assist and emphasize the need for households to produce food to address household food insecurity and hunger. In the same light, the objective of the then Department of Agriculture, Forestry and Fisheries (DAFF) (now renamed the Department of Agriculture, Land Reform and Rural Development) was to develop agricultural policies and initiate projects that would ensure South Africans could produce their food to be food secure. This objective is closely aligned with the Food and Nutrition Security Implementation Plan. Both are fundamental for achieving Goal 2 of the Sustainable Development Goal: ending hunger and achieving food security and nutrition [17,18].

For context-appropriate policies and programs to be implemented that enhance home gardening for rural dwellers, there is a need to first determine how home gardens contribute towards household food security as well as the gaps and opportunities that may present themselves. In the past, South Africa implemented the One Home One Garden initiative, to encourage and equip households predominantly in rural areas to participate in cultivating their own food, hoping to alleviate hunger and reduce food insecurity [19]. Rammohan et al. [9] examined the role of home gardens in alleviating household food insecurity and improving dietary diversity among rural households in Myanmar. Saediman et al. [20] investigated the contribution of home food gardening to household food security in Indonesia. In addition, Issahaku et al. [21] examined farmers’ decisions to engage in subsistence home gardening and its impact on food and nutrition security among farm households in Rwanda. Although studies were conducted internationally, they demonstrate the gap in the South African context relating to home food gardens and their contribution to household food security. In light of this background, this study aims to identify the various ways that home gardens contribute towards reducing household food insecurity in Limpopo Province, South Africa.

## 2 Methodology

### Description of Study Areas

**Figure 1:**
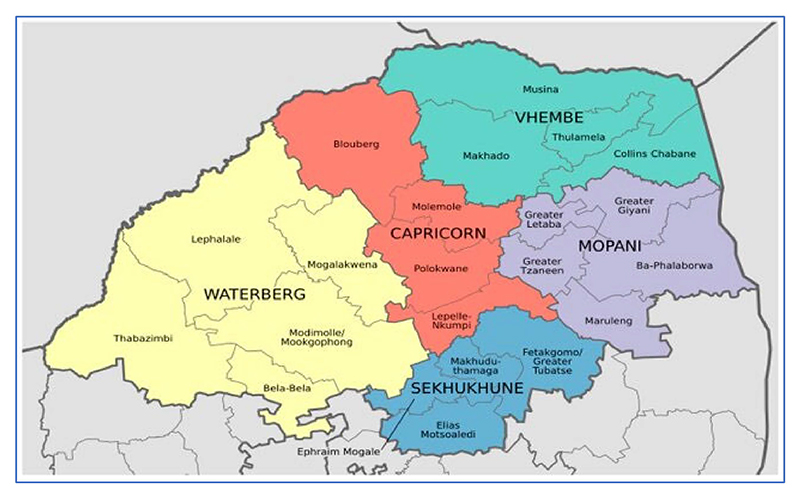
Map of demarcation district municipalities in Limpopo province.

This study used secondary data collected in Limpopo in 2021. Limpopo is one of the most resource-poor provinces in South Africa [22,23]. The gender composition structure is skewed towards the youth population where there are increasing population numbers among infants, teenagers and youth [24]. Data were collected in three areas within Limpopo province: Lobowa, Ganzankulu and Venda, shown in Figure 1 below. The total provincial population is 6.015 million people, 1.641 million households. The province has the third highest Human Development Index (HDI) in South Africa, 0.710 [25]. More than 2 million people were recorded to be unemployed in 2017, the majority being the youth, which highlights the need to increase labor force participation and job opportunities [24,26]. Most residents live in rural areas, which have given rise to an abundance of (privately owned) housing developments over a short period of time as the province is now known as having the highest percentage of people living in formal housing in the country.

Limpopo is known for its diversified agricultural activities. Cattle farming, staple crops such as maize and sunflowers, cotton, and tropical fruits are grown for domestic sales and international export. Water supply is the main problem facing the agricultural sector, as well as households. To address this challenge, many drill boreholes within their premises for water accessibility [27].

## 3 Data Collection

A total of 2043 respondents were surveyed, using structured questionnaires, to understand the status of food and nutrition security of participating households. Information collected included demographics and household welfare data. The information used in this study was extracted to obtain a comprehensive understanding of the contribution of home gardening production and household food security. This study used a quantitative research approach and the multistage stratified random sampling technique for the selection of household heads from different municipalities and districts.

## 4 Data Analysis

The quantitative data were analyzed using STATA statistical software (version 13) and Statistical Software for Social Sciences (SPSS) version 24. A descriptive statistics analysis was performed to compare the sample population’s socio-economic factors and food security status between rural households participating in home gardening production. The food security assessment used the internationally accepted food measurement tool, the Household Food Insecurity Access Scale (HFIAS).

The HFIAS evaluates the level of food insecurity (access) [28]. The HFIAS has nine questions based on an individual’s food access uncertainty and anxiety. A coded frequency for each of the nine occurrence questions related to household-level food access was used to determine the HFIAS score for each household. The maximum score for each of the nine questions is 3, while the total score, when added together, has a maximum of 27, and a minimum score of 0. The scores indicate how secure the household’s access to food is, with a higher score indicating greater food insecurity and a lower score indicating greater food security [28]. The HFIAS has four categories: secure, mild, moderate and severe.

### Endogenous Switching Poisson Regression Model

Following other empirical studies [29,30], this study used the endogenous switching Poisson regression model to assess the contribution of home gardens to household food security. The model was designed by Terza [31] and Miranda [32], who stated that given the *i*th household from a random sample *I* = {1….*n*} conditional on a vector of explanatory variables X_i_, an endogenous dummy *C*_i_ and a random term *ε*_i_, the dependent variable Y_i_, which is a count, is supposed to follow a standard Poisson distribution as follows:

(1) $$\text{F}\left({\text{Y}}_{i}/\varepsilon_{\text{i}}\right)=\frac{\text{exp}\left\{-\left({\text{x}}_{i}\text{′}\beta +{\gamma \text{C}}_{\text{i}}\varepsilon_{\text{i}}\right)\right\}{\{\text{exp}\left({\text{x}}_{i}\text{′}\beta +{\text{C}}_{i}\varepsilon_{\text{i}}\right)\}}^{\text{yl}}}{{\text{Y}}_{i}}$$

where *β* and *γ* are coefficients to be estimated. The error term *ε*_i_ measures omitted, as are unobserved variables as well as any measurement error. Given a vector of explanatory variables Z_i_ (which may contain some or all elements of) X_i_, C_i_ is characterized by an index process:

(2) $${\text{C}}_{i}=\{\begin{array}{c} 1if{\text{Z}}_{i}\alpha +V_{\text{i}}>0\\ 0otherwise \end{array}$$

where *α* is a vector of coefficients to be estimated. Suppose that W_i_ represents all endogenous variables and *ε*_i_ and *V*_i_ are jointly normal with mean zero and covariance matrix $\text{Σ}=(\begin{array}{c} \sigma^{2}\sigma \rho \\ \sigma \rho 1 \end{array})$, given that *ε*_i_, C_i_ and Y_i_ are independent. Hence, the joint conditional probability density function of Y_i_ and C_i_, given W_i_, can be written as

(3) $$F=\left({\text{Y}}_{i}/{\text{C}}_{i}/{\text{W}}_{i}\right)=\int_{-\infty }^{\infty }\left\{{\text{C}}_{i}\text{f}\left({\text{C}}_{i}/{\text{Y}}_{i}=1,{\text{W}}_{i},\varepsilon_{\text{i}}\right)Pr\left({\text{C}}_{i}=0/{\text{W}}_{i},\varepsilon_{\text{i}}\right)\right\}f\left(\varepsilon_{\text{i}}\right){\text{C}}_{i}\varepsilon_{\text{i}}$$

where *f* (*ε*_i_) denotes the probability density function for the random term *ε*_i_.

## 5 Results and Discussion

The aim of this study was to analyze the association of home gardens with household food security, with a particular focus on the rural areas of Limpopo province. This is important as it places a particular focus on one geographical region that has unique characteristics and needs that could differ in comparison with other provinces in South Africa. This section discusses the results obtained from the HFIAS as a standard food security measurement tool and the quantitative data analyzed using STATA and SPSS.

### 5.1 Descriptive Results

This study involved 2043 rural households, of which 53.8% were female and 46.2%, were male. The minimum age of the household head was 18 years old, the maximum was 103 years old and the mean was 53.9 years old. Women were seen as the dominant gender in home gardening. Table 1 below displays the demographic characteristics of households. This may imply that they have a responsibility to care for the family while ensuring there is sufficient food for the family’s needs, especially for those unable to find formal employment. This study finding was consistent with the findings of Oguttu et al. [33] and Bhandari et al. [34], who found rural women more involved in home food gardening compared to their male counterparts. Adeosun et al. [35] also found that female-headed households positively influenced households’ home gardening and food accessibility, more than male-headed households. (Table 1).

The average age of the participants was 53 years old; older individuals participated more in home gardening production than younger persons, and the average household had an average of five members. Older study participants were more likely to participate in home gardening than younger participants. Schooling is compulsory for children between the ages of 7 and 15 in South Africa. Regarding primary and secondary educational levels, the results revealed that 27.3% had a primary level, 42.2% had a secondary level and 18.2% had no schooling, while a lower proportion of participants obtained post Matric qualifications. The lower the household’s educational level, the less probability of home garden participation. Educational status plays an important role as decisions made are influenced by knowledge of home gardening and its intended benefits for the family. For example, those with more knowledge about nutritional and economic benefits may contribute towards increased participation and food security attainment [14]. Akerele et al. [36] emphasized similar findings where household members with formal education participated in home gardens more, in comparison with those with informal education.

The quantitative data showed that 70.7% of home garden farmers were unemployed, suggesting that most rural households had a significant reliance on home gardening for food and income sources. The high unemployment rate in Limpopo was felt mostly in women-headed households, as detailed in a study by Kongolo [23] and a case study by Wanka [26]. Wanka found most household heads to be unemployed without any source of income, which is also evident in this current study. These findings are characteristic of the statistics that list Limpopo as having the third highest unemployment rate of 49.9%, after North West and Eastern Cape [37].

### 5.2 Occurrence of Household Food Insecurity Based on HFIAS Categories

**Figure 2:**
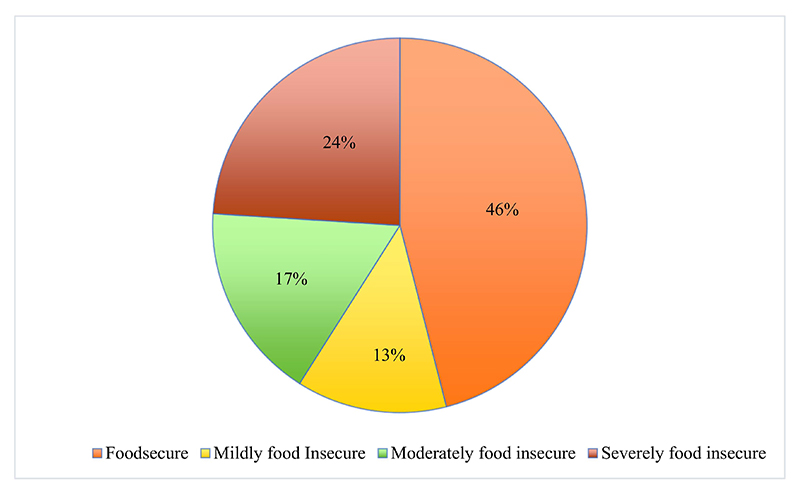
Pie chart showing the Household Food Insecurity Access Scale results.

Figure 2 illustrates the occurrence of household food insecurity which was determined using the Household Food Insecurity Access Scale (HFIAS). The findings indicated that almost 46% of households were food secure and 13% were mildly food insecure. The results also showed that 17% of households were moderately food insecure, while 24% of households were severely food insecure. These findings indicate that 54% of households faced difficulties in accessing food. Similar findings were reported in a study conducted by Sibathu and Qaim [38], where smallholder farmers were found to be food insecure.

Poverty and food insecurity affect mainly Black Africans, and many are located in rural areas where food insecurity thrives [39,40]. In 2001, more than two-thirds of impoverished households were located in rural areas where food insecurity peaked [40,41]. While the current study takes place almost two decades later, this research reveals minimal changes in terms of the food insecurity experienced by households in the country.

### 5.3 The Contribution of Home Garden Participation to Household Food Security—Endogenous Switching Poisson Regression Model

The estimated results of the endogenous switching regression model are presented in Table 2 below. A selection bias was detected and represented by the significant correlation coefficients of the selection equations. The significant results of the sigma (σi), rhos (p) and athrho indicated self-selection problems. A positive athrho value indicates that some unobservable factors affected home gardening participation. However, the endogenous switching regression model was used to address the endogeneity issue. The first stage of the endogenous switching regression analysis (the selection model) estimated the household determinants of home garden participation. The second stage estimated the effect of household determinants on the food security of home garden participants and non-participants.

Gender plays a critical role in home garden production, resulting in a positive contribution towards household food security. The current study established that the gender of the household head had a positive and significant impact on the food security status of the home garden participants. This means that females participated more in home gardening. These findings are consistent with the findings from [42], which showed women participated more in communal homes than males. Gbedomon et al. [43] also found women were more associated with home food gardens than men. Globally, women are renowned for their effective roles in agriculture as they contribute meaningfully to ensuring food security in various households. The studies further explained that women are also caregivers; thus, participating at home could be one major way in which women see themselves contributing towards food accessibility for their families, especially in times of food shortages brought about by lack of income. Although women play an important role in home gardens by ensuring food access for their families, the unequal distribution of resources in comparison to men makes women and children more susceptible to poverty and food insecurity.

The age of the household head had a negative and significant (*p*-Value 0.10) impact on the food security status of home garden non-participants. This finding implies that as the age of the household heads increases, their level of participation in home garden production decreases, which leads to food insecurity. Youth tend to have negative [44] and, at times, varying perceptions and aspirations regarding agriculture [44,45]. These mixed perceptions may negatively impact households, especially with youthful members not interested in agricultural activities due to preconceived social misconceptions and a lack of skills. On the contrary, study results from Gbedomon et al. [43] showed a significant proportion of adults and mature-aged people participating at home. The age disparity further emphasizes the need to involve younger people in the cultivation of food and the home garden value chain.

The inception of poverty is seen in a household’s inability to meet their basic needs such as finances to secure food items and other necessities. The results showed employment status had a significant impact on the food security of both home garden participants and non-participants, which was also found in studies by Mishra et al. [46] and Reardon [47], who emphasized the importance of income on household food security. The employment status had a positive impact on the food security status of the non-participants, while it showed a negative impact on the food security of home garden participants. This implies that households that did not participate in home gardening production were able to use earnings to buy food items, which contributed to being food secure, while households that participated in home gardens were negatively impacted by lack of employment brought on by lack of income for food items, thus negatively impacting their food security. Anderson [48] found that households produced their own food for consumption inadequate for meeting the household’s dietary needs which led to the increased need for off-farm employment to aid in accessing food items, while Mishra et al. [46] documented a positive relationship that was found between employment and food security—showing similar findings to this current study. Additional research is required to address the nuances and complexities regarding the concept of food self-sufficiency in relation to home gardens.

Household size showed a significant relationship with home garden participants, which implies that household size may contribute to more garden participation, further contributing to food accessibility. Other studies have revealed household size to be one of the determinants of food security [49,50]. Dzanku [51] shared that smallholder farmers have a large dependency on their own household members for on-farm or off-farm income generation. Similar to the findings of the current study, Aidoo et al. [52] concluded household size is among the factors that affect food security, where the probability of a household being food secure increased with household size, while Sikwela [53] showed contradicting findings where household size was found to influence household food insecurity significantly due to increase in the demand for food.

Increasing home food production alone might not provide sufficient steady supplies for prolonged periods. This is where additional income plays a critical role. Working for a wage had a positive and significant impact on the food security of home garden participants at a level of 1%. However, it had a negative impact on the food security of home garden non-participants. This shows that home garden participants were also working to earn supplemental income, to buy other types of food outside of home gardens. On the contrary, home garden non-participants who worked for a wage or salary had increased chances of being food insecure, possibly due to the sole reliance on purchased foods, without access to home garden produce for additional food accessibility. Dzanku [51] found that when off-farm income is obtained by household members, the average food availability is increased. This in turn decreases the chances of those households experiencing food insecurity.

Social relief funds had a negative impact and statistically influenced the food security status of home garden participants. Ntombela et al. [54] noted how South Africa has a large population (20%) that cannot feed itself, due to insufficient resources. Similar findings were documented by Chakona and Shackleton [55,56] by comparing households that received social grants and those that did not: households that were more food insecure were those receiving social grants or relief. Ideally, receiving social relief funds is associated with increased food security, contrary to the findings of this study. To understand the food insecurity dynamics for households in rural households in Limpopo, these findings suggest further qualitative investigation is required to ascertain detailed reasons as to how and why social relief funds have a negative impact on household food security.

South Africa has historical backdrops of socio-economic and natural resource inequality, which remains a reality in current times. Access to land plays a significant role in agricultural participation. The findings of this study showed access to land had a negative impact on home garden participation and food security of non-home garden participants. However, it indicated a positive result for the food security of home garden participants, which implies that households with minimal or no access to home gardening land were limited in their ability to participate, which negatively affected their food security. Those who participated could utilize the land they had access to produce and contribute towards their food security. Findings from Onomu et al. [14] revealed that many participants did not grow certain crops due to a lack of available land. Land inaccessibility, especially among women, inhibits food production activity. Land accessibility challenges are not unique to South Africa. In Kenya, the inheritance and ownership of land by women are greatly challenged due to various customary and statutory land tenure systems, which adversely affect women’s land rights and land usage, thus further posing a negative impact on the food security status of households [57]. Agricultural land availability is a crucial determinant of household food as it directly correlates with land usage activities [14,58]. Menon et al. [59] further conclude that women’s land ownership yields beneficial results for the household and contributes to well-being and higher status.

Agriculture-related assistance results showed a negative and significant impact on home garden participation and a positive influence on the food security of home garden participants. Food insecurity is brought about by various factors among rural households, which can be remedied to a certain degree with the facilitation of agriculture-related assistance. This may be in the form of agricultural training, input supplies, availability of extension workers, financial support and resource allocation directed at improving home garden farming. The current study has shown how important agricultural assistance is in improving household food security, especially encouraging home gardening in Limpopo, similar to Oladipo and Grobler’s [60] finding on the importance of agriculture-related assistance to home in South Africa as this contributes towards household food security through increased food production.

Lastly, the results showed that market distance had a positive and significant impact on the food security of home garden participants. Market accessibility impacts rural households’ food security more as farmers are both manufacturers and users [61]. Farmers sell their products at markets and buy food and other items for their sustenance [62]. When home gardens reach harvesting season, their produce often exceeds quantities of food usually purchased from the market, providing food access and profits. The findings of this study confirmed the importance of market distance in ensuring household food security. Factors such as long distances may lead to transportation costs related to rising fuel prices, which may hinder market accessibility and inability to sell produce by home garden farmers. Munawar et al. [61] also state how expensive transportation costs have contributed towards restricting food access due to the domino effect on food prices.

### 5.4 Treatment Effects of Impact of Home Garden Participation on Household Food Insecurity

This research aimed to see how home garden participation affected household food security. A simple significant difference in food security between non-home garden participants and participants can be biased and fails to account for potential heterogeneity in the two groups’ characteristics. As a result, this study used average treatment effects (ATEs), average treatment effect on the treated (ATT) and average treatment effect on the untreated (ATU) to compare the potential outcomes. Findings from treatment effects are presented in Table 3. The ATT had a positive sign, but it was not significant, which means that home garden participants did not have any considerable effect on the food insecurity status of households. According to the ATE estimate, the average smallholder farmer who participated in the home garden production had improved food security, as results showed that home gardens had a negative and significant (*p*-Value < 0.05) effect on household food insecurity. The results are also similar for the ATU estimates, which showed negative and significant results for household food insecurity.

## 6 Conclusions

This study brings to light the critical role that home gardening plays in addressing food security challenges in rural communities, as shown by the key findings from this study conducted in Limpopo Province. The high prevalence of food insecurity among households highlights the urgent need for implementing effective interventions.

This study found factors such as gender, household size, access to land and proximity to markets were major influencers of household food security. Importantly, this study showed a positive impact on food security among households that participated in home gardening.

To further enhance food security and promote sustainable livelihoods in rural communities, targeted interventions are required. Agricultural training and skill-building programs can potentially empower individuals to effectively engage in home gardening, thus increasing access to nutritious food and supplementary income. In addition, the integration of agricultural curricula can foster a systematic and deeper understanding of food systems from a young age, providing a foundation for informed decisions about food production and consumption within households. By the identification of the significance of home gardening in rural communities by policymakers and stakeholders, the challenge of food insecurity can be directly addressed.

## Figures and Tables

**Table 1:** Demographic characteristics of Limpopo households.

Characteristics	%
Gender	
Male	46.2
Female	53.8
Participant household distribution by study site	
Capricorn	18.7
Greater Sekhukhune	21.5
Mopani	21.2
Vhembe	20.8
Waterberg	17.8
Educational Level	
Primary School Education	27.3
Secondary School Education	42.2
National Training Certificate (NTC) level 1–3	1.7
National Training Certificate level 4–6	0.8
Diploma with less than Matric	0.4
Higher/national/advanced certificate with Matric	0.6
Diploma with Matric/occupational certificate	2.1
Higher Diploma/occupational certificate (b-tech diploma)	1.2
Post Higher Diploma (Masters Diploma/Degree)	0.2
Bachelor’s Degree/occupational certificate	1.5
Honours degree/postgraduate diploma/occupational certificate	0.4
Doctoral degrees (doctoral diploma and Ph.D.)	0.2
No schooling	18.2
Employment Status	
Employed full-time	15.4
Employed part-time	7.0
Self-employed	5.8
Not employed	70.7
Studying full-time	1.1
Relationship status	
Living in common-law marriage	38.0
Cohabiting	10.8
Divorced	1.8
Legally separated	0.6
Spouseless	20.9
Single	27.9
Salaries and wages	23.2
Household income (ZAR)	
No income	1.5
Less 1500	21.7
1501–3000	38.9
3001–4500	18.6
4501–6000	6.5
Greater than 6000	12.9
Access to land	36.0
Own land	88.9
Rent land	1.1
Tribal authority	3.5
State-owned land	0.6
Other	6.0
Land used for the production of food and other agricultural products	82.5

**Table 2:** Impact of home garden participation on household food insecurity—endogenous switching Poisson regression model.

Status	Home Garden Participation			Food Security of Participants			Food Security of Non-Participants		
	Coefficient	Std. Err	*p*-Value	Coefficient	Std. Err	*p*-Value	Coefficient	Std. Err	*p*-Value
Gender of household head	0.161	0.104	0.122	2.164	0.064	0.000 ***	−0.028	0.051	0.583
Age of household head	−0.003	0.003	0.327	0.000	0.001	0.817	−0.003	0.001	0.065 *
Employment status	−0.018	0.059	0.761	−0.040	0.013	0.002 ***	0.090	0.026	0.001 ***
Household size	0.048	0.020	0.016 **	0.009	0.004	0.046 **	−0.001	0.009	0.950
Did work for a wage/salary	−0.041	0.128	0.751	0.083	0.032	0.009 ***	−0.192	0.064	0.003 ***
Receive any social relief	0.069	0.115	0.551	−2.109	0.063	0.000 ***	0.086	0.052	0.100
Access to land	−3.323	0.228	0.000 ***	0.114	0.034	0.001 ***	−1.690	0.471	0.000 ***
Agriculture- related assistance	−0.743	0.290	0.010 **	2.483	0.149	0.000 ***	−0.092	0.095	0.334
Education	0.001	0.002	0.497	−0.000	0.000	0.705	−0.002	0.001	0.035 **
Marital status of household head	0.013	0.022	0.555	0.004	0.005	0.505	0.004	0.011	0.711
Market distance	0.004	0.006	0.578	0.008	0.002	0.000 ***	−0.023	0.017	0.191
_cons	4.908	0.720	0.000 ***	−3.660	0.330	0.000 ***	1.436	0.585	0.014 **
/lnsigma0	1.242	0.026	0.000						
/lnsigma1	1.235	0.065	0.000						
/athrho0	0.078	0.005	0.000						
/athrho1	0.107	0.036	0.000						
sigma0	3.461	3.289	3.642						
sigma1	3.437	3.024	3.906						
rho0	−0.147	0.005	0.000						
rho1	−0.172	0.036	0.000						
Log likelihood	−5272.316								
Prob > chi2	0.000								
Wald chi2(11)	1300.68								

* Indicates significance at 5% level.

** Indicates significance at 1% level

*** Indicates significance at 0.01% level.

**Table 3:** Treatment effects of the impact of home garden participation on household food insecurity.

	Estimate	SE	*p*-Value
ATE (average treatment effect)	−236.38	105.66	0.025
ATT (average treatment effect on the treated)	−69.053	187.52	0.712
ATU (average treatment effect on the untreated)	−278.44	100.39	0.005
LATE (local average treatment effect)	−958.91	615.75	0.119

## Data Availability

The data presented in this study are available on request from the corresponding author.

## References

[1] Galhena D, Freed R, Maredia K (2013). Home Gardens: A Promising Approach to Enhance Household Food Security and Well Being. Agric Food Secur Agric Food Secur.

[2] IISD World Population to Reach 9.9 Billion by 2050 https://sdg.iisd.org/news/world-population-to-reach-9-9-billion-by-2050/.

[3] Adekunle O (2013). The Role of Home Gardens in HouseholdFood Security in Eastern Cape: A Case Study of Three Villages in Nkonkobe Municipality. J Agric Sci.

[4] Agriculture and Economic Development Analysis Division (2012). The State of Food Insecurity in the World.

[5] Stats SA How COVID-19 Affected Food Security in South Africa.

[6] GFSI (2022). Global Food Security Index.

[7] Cerda C, Guenat S, Egerer M, Fischer L (2022). Home Food Gardening: Benefits and Barriers During the COVID-19 Pandemic in Santiago, Chile. Front Sustain Food Syst.

[8] Ferreira ADJ, Marquez R, Santos C, Martins M (2018). Urban Agriculture: A Tool Towards More Resilient Urban Communities?. Environ Sci Health.

[9] Rammohan A, Pritchard B, Dibley M (2019). Home Gardens as a Predictor of Enhanced Dietary Diversity and Food Security in Rural Myanmar. BMC Public Health.

[10] Tesfamariam BT, Owusu-Sekyere E, Emmanuel D (2018). The Impact of the Homestead Food Garden Programme on Food Security in South Africa. Food Secur.

[11] Vavra J, Megyesi B, Duzi B, Craig T, Klufova R, Cudinova E (2018). Food Self-provisioning in Europe: An Exploration of Sociodemographic Factors in Five Regions. Rural Sociol.

[12] Korpelainen H (2023). The Role of Home Gardens in Promoting Biodiversity and Food Security. Plants.

[13] Musotsi AA, Sigot AJ, Onyango MOA (2008). The Role of Home Gardening in Household Food Security in Butere Division of Western Kenya. Afr J Food Agric Nutr Dev.

[14] Onomu AR, Aliber M, Tarivunga A, Chinyamurindi WT, Megbowon ET (2022). Drivers of Home Garden Growth beyond Food Security and Income: Lessons from South Africa. Int J Dev Sustain.

[15] Department of Justice and Constitutional Development (1996). The Constitution of the Republic of South Africa.

[16] Masipa TS (2017). The Impact of Climate Change on Food Security in South Africa: Current Realities and Challenges Ahead. Jamba.

[17] Department of Agriculture Forestry and Fisheries (2013). National Policy on Food and Nutrition Security.

[18] Statistics South Africa (2019). SDG Country Report.

[19] Nesengani T, Mudau M, Netshandama V (2016). Contribution of Food Security Projects on Poverty Alleviation to the communities of Limpopo Province, South Africa. S Afr J Agric Ext.

[20] Saediman H, Gafaruddin A, Hidrawati H, Salam I, Ulimaz A, Rianse IS, Taridala SAA (2021). The Contribution of Home Food Gardening Program to Household Food Security in Indonesia: A Review. WSEAS Trans Environ Dev.

[21] Issahaku G, Kornher L, Islam AH, Abdul-Rahaman A (2023). Heterogeneous Impacts of Home-Gardening on Household Food and Nutrition Security in Rwanda. Food Secur.

[22] Gyekye AB, Akinboade OA (2003). A Profile of Poverty in the Limpopo Province of South Africa. EASSRR.

[23] Kongolo M (2009). Women in Poverty: Experience from Limpopo Province, South Africa. Afr Res Rev.

[24] Provincial Treasury (2019). Limpopo Socio-Economic Review and Outlook.

[25] Global Data Lab Sub-National HDI Area Database.

[26] Wanka FA (2014). The Impact of Educational Attainment on Household Poverty in South Africa: A Case Study of Limpopo Province, PhD Thesis.

[27] Molele C (2016). Mail & Guardian.

[28] Coates J, Swindale A, Bilinsky P (2007). Household Food Insecurity Access Scale for Measurement of Food Access: Indicator Guide.

[29] Donkoh S (2020). Rice Commercialization and Improved Agricultural Technology Adoption in Northern Ghana: Endogenous Switching Poisson Approach. Alanya Akad Bakis.

[30] Hasebe T (2020). Endogenous Switching Regression Model and Treatment Effects of Count-Data Outcome. Stata J.

[31] Terza VJ (1998). Estimating Count Data Models with Endogenous Switching: Sample Selection and Endogenous Treatment Effects. J Econom.

[32] Miranda A (2004). FIML Estimation of an Endogenous Switching Model for Count Data. Stata J.

[33] Oguttu JW, Mbombo-Dweba TP, Ncayiyana JR (2021). Factors Correlated with Home Gardening in Gauteng Province, South Africa. Int J Environ Res.

[34] Bhandari S, Yadav P, Rijal S (2021). Home Garden; an Approach for Household Food Security and Uplifting the Status of Rural Women: A Case Study of Saptari, Nepal. Turk JAF Sci Technol.

[35] Adeosun KP, Nnaji AP, Onyekigwe CM (2020). Socio-Economic Determinants of Home Gardening Practices among Households in University of Nigeria Community: Heckman Double Stage Selection Approach. J Trop Agric Food Environ Ext.

[36] Akerele D, Awoyemi S, Sanusi RA, Ibrahim SB (2017). Effects of Household Home Garden, Socioeconomic Characteristics and Health Status Perception on Food Consumption Diversity in Oyo State, Nigeria. FUW Trends Sci Technol.

[37] Statistics South Africa (2022). Quarterly Labour Force Survey Q4.

[38] Sibhatu KT, Qaim M (2017). Rural Food Security, Subsistence Agriculture, and Seasonality. PLoS ONE.

[39] De Cock N, D’Haese M, van Rooyen CJ, Schönfeldt HC, D’Haese L (2013). Food Security in Rural Areas of Limpopo Province, South Africa. Food Sec.

[40] Ramkissoon Y (2022). SA’s Rural Areas and Smaller Municipalities Need National Support to Tackle Poverty.

[41] Watkinson E, Makgetla N South Africa’s Food Security Crisis. NALEDI.

[42] Nzama N, Ntini E (2022). Challenges Facing Women’s Community Vegetable Gardening in the Echobeni Area of KwaZulu Natal Province, South Africa. Afr J Gend Soc Dev.

[43] Gbedomon RC, Fandohan AB, Salako VK, Idohou AFR, Kakai RG, Assogbadjo AE (2015). Factors Affecting Home Gardens Ownership, Diversity and Structure: A Case Study from Benin. J Ethnobiol Ethnomedicine.

[44] Mibey MC (2015). Factors Influencing Youth Involvement in Agribusiness Projects in Bomet Central Sub-County, PhD Thesis.

[45] Chipfupa U, Tagwi A (2021). Youth’s Participation in Agriculture: A Fallacy or Achievable Possibility? Evidence from Rural South Africa. S Afr J Econ.

[46] Mishra AK, Mottaleb KA, Mohanty S, Mishra AK, Mottaleb KA, Mohanty S (2015). Impact of off-Farm Income on Food Expenditures in Rural Bangladesh: An Unconditional Quantile Regression Approach. Agric Econ.

[47] Reardon T (1997). Using Evidence of Household Income Diversification to In-Form Study of the Rural Nonfarm Labor Market in Africa. World Dev.

[48] Anderson AS (2002). The Effect of Cash Cropping, Credit, and Household Composition on Household Food Security in Southern Malawi. Afr Stud Q.

[49] Babatunde RO, Quaim M (2011). Impact of Off-Farm Income on Food Security and Nutrition in Nigeria. Food Policy.

[50] Oluwatayo IB, Ojo AO (2019). Effect of Access to ICT on Food Insecurity among Farming Households in Nigeria. J Dev Areas.

[51] Dzanku FM (2019). Food Security in Rural Sub-Saharan Africa: Exploring the Nexus between Gender, Geography and off-Farm Employment. World Dev.

[52] Mensah JO, Aidoo R, Tuffour T (2021). Determinants of Household Food Security in the Sekyere-Afram Plains District of Ghana. Glob J Plant Soil Sci.

[53] Sikwela M (2008). Master’s Thesis.

[54] Ntombela S, Bohlmann H, Kalaba M (2019). Greening the South African Economy Could Benefit the Food Sector: Evidence from a Carbon Tax Policy Assessment. Env Resour Econ.

[55] Chakona G, Shackleton CM (2017). Voices of the Hungry: A Qualitative Measure of Household Food Access and Food Insecurity in South Africa. Agric Food Secur.

[56] Chakona G, Shackleton CM (2019). Food Insecurity in South Africa: To What Extent Can Social Grants and Consumption of Wild Foods Eradicate Hunger?. World Dev Perspect.

[57] Owoo NS, Boakye-Yiadom L (2014). The Gender Dimension of the Effects of Land Tenure Security on Agricultural Productivity: Some Evidence from Two Districts in Kenya. J Int Dev.

[58] Mutangadura G (2004). Proceedings of the Gender.

[59] Menon N, Rogders Y, Kennedy AR (2016). Land Reform and Welfare in Vietnam: Why Gender of the Land-Rights Holder Matters. J Int Dev.

[60] Oladipo OD, Grobler W (2022). Status Quo of Households’ Backyard Gardens in South Africa: The “Drivers“. Sustainability.

[61] Munawar M, Shiwei X, Wen Y, Muhammad L (2021). Investigating Relationship of Food Security with Market Approachability with Respect to Household Food Insecurity Index. J Econ Impact.

[62] Ahmed UI, Ying L, Bashir MK, Abid M, Elahi E, Iqbal MA (2016). Access to Output Market by Small Farmers: The Case of Punjab, Pakistab. J Anim Plant Sci.

